# The problematic use of cultural symbols on Chinese cigarette packs

**DOI:** 10.18332/tid/162301

**Published:** 2023-05-19

**Authors:** Emily Xing, Zheng Dai, Alena Madar, Kevin Welding, Katherine Clegg Smith, Joanna E. Cohen

**Affiliations:** 1 Institute for Global Tobacco Control, Johns Hopkins Bloomberg School of Public Health, Baltimore, United States; 2 Department of Economics, The George Washington University, Washington, United States

**Keywords:** China, cigarettes, smoking, marketing, policy

## Abstract

**INTRODUCTION:**

China is the world’s largest tobacco-consuming nation. With minimal packaging regulations, the Chinese tobacco industry can use many appeals to promote their products, including calling upon traditions and culture to make positive connections between consumers and harmful products. We analyzed the nature and extent of cultural appeals on Chinese cigarette packs.

**METHODS:**

A total of 610 unique cigarette packs were collected in 2017 from five major Chinese cities (Beijing, Guangzhou, Shanghai, Kunming, and Chengdu) following a systematic protocol. Two trained independent coders knowledgeable about Chinese culture and language coded the packs in accordance with a specially developed codebook encompassing important Chinese cultural symbols. The prevalence of identified elements was determined and interpreted.

**RESULTS:**

Overall, 60.7% (n=370) of the analyzed Chinese cigarette packs in our sample contained at least one culturally specific appeal. The most common cultural appeals included written arts (n=131; 21.5%), celebratory red as the primary pack color (n=119; 19.5%), visual arts (n=70; 11.4%), and special occasions (n=60; 9.9%). There was a diverse range of cultural appeals present on the packs.

**CONCLUSIONS:**

Cultural appeals are common on Chinese tobacco packaging, with over 60% of all analyzed packs containing at least one culturally specific element. With China’s packaging policies requiring health warning labels to occupy only 35% of the pack, the tobacco industry is allowed plenty of package space to incorporate cultural elements among other appeals. A plain and standardized packaging policy would eliminate the ability for Chinese tobacco companies to use cultural appeals on their cigarette packs.

## INTRODUCTION

China, the country with the largest tobacco burden, is home to over 300 million people who smoke with over half of the male population currently using tobacco^1^. Despite efforts to curtail tobacco use^2^, the Chinese tobacco industry is still able to attract consumers through marketing tactics that can increase smoking susceptibility^3^, particularly efforts to attract populations where smoking has traditionally been less acceptable, including youth and women.

Some tobacco control policies have been enacted by the Chinese government. In 2015, China’s National Advertising Law was updated to prohibit all tobacco marketing via mass media^4^, such as film, television, and newspapers. However, China falls significantly behind other countries with respect to its required health warning labels (text only, covering 35% of the front and back of the pack^4^) and in limiting the potential for attractive appeals and misleading information about product harm on tobacco packs^5,6^.

Research has shown that product packaging is an impactful method of communication between companies and consumers. Color, content, imagery, and lexicon are all aspects of a pack that can engage specific audiences and affect a consumer’s view of the product, such as misleading them into thinking certain tobacco products are ‘safer’^7,8^. Thus, the use of appeals on tobacco packaging may contribute to the high prevalence of smoking in China, especially given that other mediums of tobacco marketing have been restricted^9^.

As tobacco packs remain an important advertising avenue, China’s lax packaging legislation provides cigarette companies with a lot of surface area to contain attractive appeals on their products. These appeals include elements specifically targeting consumers in China, such as those pertaining to Chinese culture. Marketing initiatives for consumer goods like cigarettes can draw upon national identity elements including patriotism and topophilia (affinity to place) to create positive associations between the product and the potential consumer^10^. Tobacco companies have used cultural connections and symbols to promote smoking^11,12^. Examples of these imagery and lexical elements on cigarettes include those depicted in traditional Chinese art forms, such as brush calligraphy and ink painting^13^. There is also potential to incorporate important cultural symbols, such as those involving nature (e.g. pandas, the nation’s animal^14^, and lotuses, a traditional symbol for purity)^15^ and national landmarks (e.g. the Yellow Mountain and Forbidden City)^16,17^. China uniquely has a long-established gift-giving culture that promotes cigarettes as a social currency^18^. In Chinese tradition, presenting gifts on special occasions can help individuals maintain healthy societal relationships through indicating respect^19^; thus, popular gifts commonly possess symbols of prosperity^20^, which can be communicated on tobacco packs through the celebratory color red symbolizing joy and luck^21^, the ‘fú’, or fortune, character (福)^22^, and other similar traditional symbols of wealth. These cultural appeals promote cigarettes as a gift in China, with nearly half of all individuals in China having either gifted or received cigarettes at some point in their lifetime^23^.

The World Health Organization and Campaign for Tobacco-Free Kids^19,24^ have briefly noted the usage of famous Chinese icons and occasion-related symbols on Chinese tobacco packs that brand them for gift-giving. Literature also exists examining cultural appeals on Chinese cigarette packs relating to ceremonial weddings and health symbols^25,26^. However, there exists a gap in literature examining the nature and extent of Chinese cultural appeals on cigarette packs. To the best of our knowledge, this study is the first to comprehensively examine the range of cultural appeals on Chinese cigarette packs. The findings can inform policy interventions that prevent the use of Chinese traditions and culture to make positive connections between consumers and these harmful products.

## METHODS

### Study sample

The ongoing Tobacco Pack Surveillance System (TPackSS) study, conducted by the the Institute for Global Tobacco Control of the Johns Hopkins Bloomberg School of Public Health (https://globaltobaccocontrol.org/tpackss/), monitors the design features, marketing appeals, and health warning label compliance of unique tobacco packs sold in low-income and middle-income countries with the greatest numbers of people who smoke^27^. In all, 738 unique cigarette packs were collected from five of the ten most populated cities in China as a part of the TPackSS study in February 2017. These sample cities (Beijing, Guangzhou, Shanghai, Kunming, and Chengdu) were selected based on population size and cultural, geographical, religious, and linguistic diversity across China. Data collectors followed a systematic walking protocol over several days to collect unique cigarette packs from a sample of vendors in neighborhoods of varying socioeconomic strata (high-, middle-, and low-income) in each city. Neighborhoods were selected based on census and property value data, for a total of 12 neighborhoods visited in each city and 60 neighborhoods total in China.

Cigarette packs were purchased from four types of vendors that included convenience stores, supermarkets, tobacco and alcohol specialty shops, and ‘mom-and-pop’ shops (small, independent, family-owned businesses). Vendor types were selected using national surveillance data on tobacco product sources and discussion with key tobacco control informants. The first visited vendor served as an index vendor for all other stores in which all unique packs were purchased and archived in an image database. A pack was considered unique if it contained at least one novel exterior pack feature including stick count, size, brand name, colors, and inclusion of a promotional item (a different health warning label did not count as a new feature). Up to five vendors were then visited in the remaining neighborhoods to collect and record any unique packs that were not already purchased. A detailed description of the standardized TPackSS methodology can be found in the published protocol by Smith et al.^27^.

### Coding

A total of 610 purchased packs contained a mainland Chinese government health warning label, indicating its legality and intended sale in the country. We excluded illicit packs from outside of China including special regions, such as Hong Kong. Using the image archive published on the TPackSS site (https://globaltobaccocontrol.org/tpackss/China), the selected sample of packs was assessed in accordance with a specially developed codebook designed based on literature presenting the most prevalent aspects of Chinese culture. The most common of these aspects were noted after observing a breadth of the sample packs, making them the focus appeals. Elements coded for inclusion were: 1) Celebratory red as the primary pack color; 2) Nationalism/patriotism; 3) Animals; 4) Plants; 5) Occasions; 6) Mythology/prosperity; 7) Food/herbs; 8) Prosperity/superstition; 9) Apparel/beauty; 10) Architecture; 11) Visual arts; 12) Written arts; 13) Performing arts; and 14) Other cultural appeals not belonging to any previous category. Although each appeal category was well-defined, most cultural appeals included a diverse range of imagery and lexicon that were found on the packs. For instance, observed lexical styles considered traditional Chinese written arts included Chinese-style seals, calligraphy, and scroll poetry. Visual arts appeals included Chinese-style painting, porcelain, and motifs/patterns. Symbols of prosperity included stone lions, gold coins, and maneki-neko lucky cats, while elements pertaining to special occasions included lanterns, messages such as ‘gonghexinx’, and wedding imagery (e.g. dual magpies). Further details about each code are in the Supplementary file.

People with Chinese backgrounds (including the first author) took the lead in developing the codebook, and two coders with Chinese backgrounds were trained extensively via multiple coding rounds before independently assessing each unique pack. The codebook underwent multiple revisions throughout the coding and training process to reflect any discussed changes. Any disagreements were resolved by a third coder without a Chinese background acting as a reviewer. Overall, the inter-rater reliability was strong to near perfect across all codes, with the agreement ranging from 86.7 to 99.7% and prevalence and bias adjusted kappa statistic ranging from 0.73 to 0.99 with an average of 0.94. Despite good agreement, all disagreements were discussed by a third reviewer. Detailed inter-rater reliability for each code can be found in the Supplementary file.

### Statistical analysis

Through descriptive analysis via Google Sheets, the frequencies of variables were determined from the set of double-coded pack data, indicating the prevalence of identified appeals within the sample.

## RESULTS

Overall, 60.7% (n=370) of the unique Chinese cigarette packs in our sample contained at least one culturally specific appeal. The most common appeals included written arts (n=131; 21.5%), use of celebratory red as the primary pack color (n=119; 19.5%), and visual arts (n=70; 11.5%). Beyond appeals that dealt with the styles in which elements were depicted, there was also a prominence of cultural symbols that were distinctly imagery or lexicon, such as representations of Chinese nationalism/patriotism (n=119; 19.5%, e.g), plants (n=46; 7.5%), animals (n=35; 5.7%), apparel/beauty (n=14; 2.3%), performing arts (n=3; 0.5%), and food/herbs (n=2; 0.3%). There were also many packs containing abundant appeals relating to cultural ideology and gift-giving, such as special occasions (n=60; 9.8%), mythology/religion (n=46; 7.5%), and prosperity/superstition (n=9; 1.5%). Table 1 shows the prevalence of each cultural appeal in order of decreasing prevalence.

**Table 1:** Prevalence of Chinese cultural appeals on unique cigarette packs, China, 2017 (N=610)

*Cultural appeals*	*n*	*%*
Traditional written arts styles	131	21.5
Celebratory red as primary pack color	119	19.5
Traditional visual arts styles	70	11.5
Special occasions	60	9.8
Nationalism/patriotism symbols	52	8.5
Mythological/religious symbols	46	7.5
Important cultural plants	46	7.5
Traditional architectural styles	40	6.6
Traditional apparel/beauty	14	2.3
Prosperity/superstition symbols	9	1.5
Cultural performing arts	3	0.5
Iconic food/herbs	2	0.3
Other appeals (e.g. science, technology)	1	0.2

**Figure 1:**
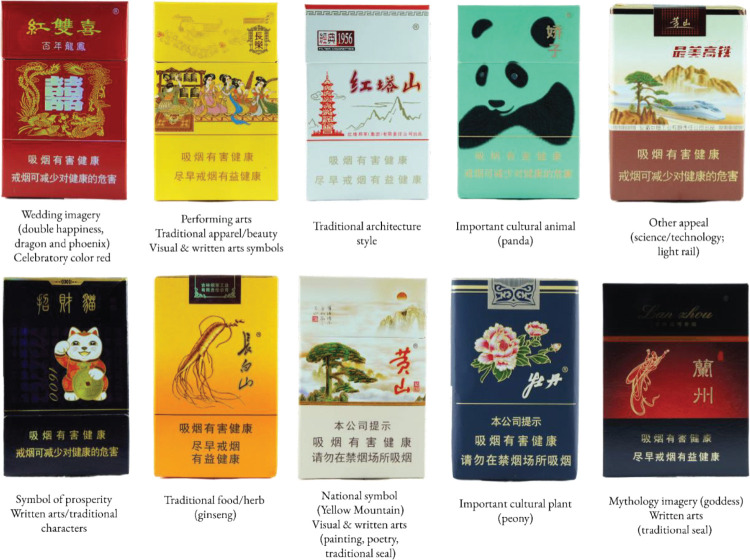
Examples of cigarette packs purchased in China that contain cultural appeal features, such as special occasions, red color, art styles, natural symbols, science/technology, prosperity, food, nationalism, and mythology, China, 2017

There was a diverse range of cultural appeals present on the packs. Figure 1 shows a variety of cultural appeals on cigarette packs.

## DISCUSSION

This study fills a gap in current tobacco packaging literature by describing the extent to which Chinese cigarette packs contain cultural appeals. We found that over half of all analyzed packs (n=370; 60.7%) contained at least one culturally specific appeal, suggesting that cultural elements are commonly used by tobacco companies as a marketing tactic that specifically targets Chinese consumers. In addition, appeals pertaining to traditional Chinese styles (e.g. usage of celebratory color red, visual and written arts) were most popular, indicating that tobacco companies are associating their products with styles, ideas and images that will be very familiar to potential consumers as a way of making harmful products seem familiar or understood or a part of a way of life. The frequency of wealth, celebration, and longevity-related elements suggests that many cigarette packs are marketed for gift-giving occasions, e.g. ceremonial weddings and Lunar New Year^18^. Other newly-explored areas of Chinese cultural appeals can potentially mislead consumers as well, representing a range of positive Chinese values from traditional idealized feminine beauty, health, longevity, and traditional architectural styles symbolizing the heavens^28,29^.

The extent and scope of varying identified cultural appeals on Chinese tobacco packs is notable and concerning. Such appeals encourage cigarette gift-giving and promote smoking as not only lower risk, but also socially acceptable. Malone^30^ explained how, through cultural appeals in general, the tobacco industry associates their products with familiar religious figures, health professionals, national landmarks and more, thereby disguising tobacco’s harmfulness and hindering effective control policy. Specifically in China, such marketing techniques may influence social attitudes about tobacco use by linking cigarettes with deeply rooted cultural meanings. This can result in further integrating smoking into Chinese society.

### Limitations

There are some limitations of this study. First, although extensive background research was conducted and the two coders were of Chinese heritage, some appeals relating to culture could have been overlooked in the coding process. The codebook may not have included every aspect of Chinese culture, only those that the co-authors identified and agreed were likely to be presented in image and lexical forms on cigarette packs. The extent to which someone may consider an appeal a cultural appeal may also vary based on geographical location or personal experience, so only the most conspicuous and agreed-upon cultural appeals were accounted for. Furthermore, some appeals possessed multiple meanings that fit more than one variable definition, but only the most specific code was selected for each unique appeal. For instance, a depiction of a dragon and phoenix is a traditional Chinese wedding symbol for prosperity^31^. Though dragons and phoenixes may separately be considered a mythology appeal, together they indicate a special Chinese cultural occasion, which is the code that would have been selected. Our study also does not compare the prevalence of cultural appeals across geographical locations, as the TPackSS study design aims to obtain a census of unique packs in a country; a unique pack is bought only the first time it is encountered. In addition, some packs possessed exterior colors and minute details that may not have been clearly observed via the pack images published on the TPackSS website. Based on these limitations, our results are likely conservative. It is important to note that TPackSS purchases unique packs, and therefore percentages do not reflect purchase patterns or market share.

### Future considerations

Future research could include coding brand names. This analysis is focused on the pack itself and therefore excluded a consideration of the brand name. It is, however, possible that tobacco companies are also positioning the names for these cigarette products in relation to traditional Chinese culture. An example of this is the brand ‘Hongtashan Gonghexinxi’ incorporating the phase ‘wish you the best’ in their brand name to promote cigarette gift-giving during special festivals^19^. Other studies could examine the use of cultural appeals on tobacco packs in other low- and middle-income countries. For instance, we have previously reported cultural appropriation on tobacco packs from Mexico^32^, such as the use of celebratory flags, colors from the Mexican flag, and ‘ofrendas’. Qualitative and/or experimental studies could investigate the influence of cultural appeals on consumer perceptions and use.

This study supports the recommendation of the World Health Organization’s Framework Convention on Tobacco Control that parties consider plain and standardized packaging to reduce the overall attractiveness of tobacco products. Plain packaging policies have been implemented in a number of countries including Australia, France, the UK, Hungary, Ireland, New Zealand, and Canada. In 2016, the Australian government published a post-policy review of this legislation and indicated that within a period of 34 months, the prevalence of tobacco use had dropped 0.55 percentage points, equivalent to over 118000 fewer people smoking^33^.

The Chinese government has not revised its tobacco packaging policies since 2007^9^. While current provisions in Chinese tobacco pack legislation prohibit explicit terms that could mislead consumers, such as ‘light’ or ‘low-tar’, they do not extend to implicit lexicon and imagery appeals that can influence social perceptions of smoking^9^. This significantly hinders China’s Healthy China 2030 goal of reducing smoking prevalence by 20 percentage points^2^.

## CONCLUSIONS

This study reveals a high prevalence and diverse range of cultural appeals on cigarette packs in China. The current state of China’s packaging and health warning label policy provides opportunities for tobacco companies to create truly beautiful packaging that makes connections between cigarettes as a product and strong and positive cultural ideas and images. Through this employment of cultural imagery and lexical appeals (e.g. traditional art forms or special occasion symbols), cigarette companies can attract, retain, and mislead consumers about a deadly product. A plain and standardized packaging policy would eliminate the ability for Chinese tobacco companies to use cultural appeals on their cigarette packs.

## Data Availability

The data supporting this research are available from the authors on reasonable request.
