# Supplementary material for: The problematic use of cultural symbols on Chinese cigarette packs
Source: Tob Induc Dis. 2023 May 19;21:65. doi: 10.18332/tid/162301 (PMC10193528; doi:10.18332/tid/162301)
Supplement: Supplementary file 1 [file TID-21-65-s1.pdf]

Supplemental Appendix: *Codebook and coding summary, with inter-rater reliability:*

Color (n=119, 19.5%) (Percent agreement=98.4%) (PABAK=0.97)

Is the primary color of the pack celebratory bright red?

(1) Yes

(0) No

Nationalism/patriotism (n=52, 8.5%) (Percent agreement=96.1%) (PABAK=0.92)

Are there images or words associated with Chinese national symbols, such as landmarks?

(e.g. Nation's flag, Champion Mao, Yellow River, Tiananmen Square, Oriental Pearl TV Tower, Yellow Mountain, Huanghelou, People's Square)

(1) Yes

(0) No

Animals (n=35, 5.7%) (Percent agreement=98.5%) (PABAK=0.97)

Are there images associated with natural animals important in Chinese culture?

(e.g. pandas, roosters, mandarin ducks, golden monkeys, cranes, koi, goldfish, magpies, tigers, butterflies, badgers, turtles, deer, or any of the twelve zodiacs)

(1) Yes

(0) No

Plants (n=46, 7.5%) (Percent agreement=98.7%) (PABAK=0.97)

Are there images associated with plants important in Chinese culture?

(e.g., bamboo, peonies, plum blossoms, red fruit trees, lotuses, chrysanthemums, willow tree, camelia, orchid, hibiscus, magnolia, azalea, narcissus, cherry blossoms, pine trees, China rose, ginkgo, jasmine)

(1) Yes

(0) No

Occasions (n=60, 9.8%) (Percent agreement=98.7%) (PABAK=0.97)

Are there images or words associated with special occasions in Chinese culture, such as holidays, weddings, birthdays, and harvests?

(e.g. lanterns, firecrackers, dragon and lion dances, red envelope, mooncake, messages such as "gonghexinxi" and "zhufu," annual harvest)

(1) Yes

(0) No

Mythology (n=46, 7.5%) (Percent agreement=97.9%) (PABAK=0.96)

Are there images or words associated with Chinese mythology?

(e.g. moon goddess, dragons, phoenixes, Monkey King, Buddha, folk tales)

(1) Yes

(0) No

Food/herbs (n=2, 0.3%) (Percent agreement=99.5%) (PABAK=0.99)

Are there images associated with traditional Chinese food or herbs/medicine?

(e.g. dumplings/dim sum, sea cucumbers, ginseng, mandarins, peaches, moon cake, zongzi, steamed buns, fried dough, tea, ginger, wolfberry, red dates)

(1) Yes

(0) No

Prosperity/superstition (n=9, 1.5%) (Percent agreement=97.5%) (PABAK=0.95)

Are there images or words associated with wealth, fortune, and luck in Chinese culture?

(e.g. yuanbao, gold coins, maneki-neko lucky cats, Chinese guardian lions)

(1) Yes

(0) No

Apparel/beauty (n=14, 2.3%) (Percent agreement=98.9%) (PABAK=0.98)

Are there images associated with traditional Chinese clothing, accessories, makeup, and other elements pertaining to beauty/appearance?

(e.g. zhongshan suit, tang suit, hanfu, qipao, armor, hairpins, futou, fans, Asian conical hat, military gear, traditional Chinese makeup such as white powder and blush)

(1) Yes

(0) No

Architecture (n=40, 6.6%) (Percent agreement=98.2%) (PABAK=0.96)

Are there images associated with traditional Chinese architecture?

(e.g. pavillion, pagoda, palace, terrace, stone bridges, roof)

(1) Yes

(0) No

Visual arts (n=70, 11.5%) (Percent agreement=86.7%) (PABAK=0.73)

Are there images depicted in traditional Chinese art styles?

(e.g. painting, porcelain, carved jade or architecture, patterns/motifs of distinctly oriental symbols such as flowers, paper cutting)

(1) Yes

(0) No

Written arts (n=131, 21.5%) (Percent agreement=92.1%) (PABAK=0.84)

Are there words depicted in traditional Chinese written art forms and styles?

(e.g. calligraphy, Chinese-style seals, up and down/right to left text orientation)

(1) Yes

(0) No

Performing arts (n=3, 0.5%) (Percent agreement=98.7%) (PABAK=0.97)

Are there images of figures engaging in traditional Chinese performing art forms, such as dance and music?

(e.g. a figure playing the erhu)

(1) Yes

(0) No

Other (n=1, 0.2%) (Percent agreement=99.7%) (PABAK=0.99)

Are there any appeals not included in other codes?

(e.g. science/technology)

(1) Yes

(0) No

© 2023 Xing E. et al.
